# Targeted TGF-βR2 Knockdown in the Retrotrapezoid Nucleus Mitigates Respiratory Dysfunction and Cognitive Decline in a Mouse Model of Cerebral Amyloid Angiopathy with and without Stroke

**DOI:** 10.21203/rs.3.rs-4438544/v1

**Published:** 2024-05-31

**Authors:** Ahmad El Hamamy, Zahid Iqbal, Ngoc Mai Le, Arya Ranjan, YuXing Zhang, Hung Wen Lin, Chunfeng Tan, Anthony Patrizz, Louise D. McCullough, Jun Li

**Affiliations:** University of Texas Health Science Center at Houston; University of Texas Health Science Center at Houston; University of Texas Health Science Center at Houston; University of Texas Health Science Center at Houston; University of Texas Health Science Center at Houston; University of Texas Health Science Center at Houston; University of Texas Health Science Center at Houston; University of South Florida; University of Texas Health Science Center at Houston; University of Texas Health Science Center at Houston

## Abstract

**Introduction:**

Cerebral amyloid angiopathy (CAA) is characterized by the deposition of amyloid-beta peptides within cerebral blood vessels, leading to neurovascular complications. Ischemic strokes result from acute disruptions in cerebral blood flow, triggering metabolic disturbances and neurodegeneration. Both conditions often co-occur and are associated with respiratory dysfunctions. The retrotrapezoid nucleus (RTN), which is crucial for CO_2_ sensing and breathing regulation in the brainstem, may play a key role in breathing disorders seen in these conditions. This study aims to investigate the role of Transforming Growth Factor Beta (TGF-β) signaling in the RTN on respiratory and cognitive functions in CAA, both with and without concurrent ischemic stroke.

**Methods:**

Adult male Tg-SwDI (CAA model) mice and C57BL/6 wild-type controls underwent stereotaxic injections of lentivirus targeting TGF-β2R2 in the RTN. Stroke was induced by middle cerebral artery occlusion using a monofilament. Respiratory functions were assessed using whole-body plethysmography, while cognitive functions were evaluated through the Barnes Maze and Novel Object Recognition Test (NORT). Immunohistochemical analysis was conducted to measure TGF-βR2 and GFAP expressions in the RTN.

**Results:**

CAA mice exhibited significant respiratory dysfunctions, including reduced respiratory rates and increased apnea frequency, as well as impaired cognitive performance. TGF-βR2 knockdown in the RTN improved respiratory functions and cognitive outcomes in CAA mice. In CAA mice with concurrent stroke, TGF-βR2 knockdown similarly enhanced respiratory and cognitive functions. Immunohistochemistry confirmed reduced TGF-βR2 and GFAP expressions in the RTN following knockdown.

**Conclusions:**

Our findings demonstrate that increased TGF-β signaling and gliosis in the RTN contribute to respiratory and cognitive dysfunctions in CAA and CAA with stroke. Targeting TGF-βR2 signaling in the RTN offers a promising therapeutic strategy to mitigate these impairments. This study is the first to report a causal link between brainstem gliosis and both respiratory and cognitive dysfunctions in CAA and stroke models.

## Introduction

Cerebral amyloid angiopathy (CAA) and ischemic stroke are significant neurological conditions that pose substantial challenges to global healthcare systems. CAA, a form of cerebral small vessel disease, is characterized by the deposition of amyloid-beta peptides within the walls of cerebral blood vessels, predominantly affecting leptomeningeal and cortical arteries [1]. This pathological accumulation leads to a spectrum of neurovascular complications, including microbleeds, cerebral infarctions, and cognitive impairments, which collectively contribute to the clinical manifestations of the disease [2–4].

Ischemic strokes, on the other hand, arise from acute disruptions in cerebral blood flow due to thrombosis or embolism, triggering immediate metabolic disturbances and subsequent neurodegeneration that result in diverse functional deficits [5, 6]. Interestingly, clinical data shows that stroke is an independent risk factor for the development of dementia [7]. The majority of dementia patients suffer from mixed brain pathologies and have histological evidence of ischemia and Alzheimer’s disease Aβ plaque accumulation, tau tangles) at autopsy [8]. Cerebral amyloid angiopathy (CAA) is also a common finding in stroke patients [9, 10].

Both CAA and ischemic stroke are increasingly recognized for their association with respiratory dysfunctions, notably sleep apnea, a condition marked by recurrent episodes of breathing cessation during sleep. Sleep apnea not only heightens the risk of cardiovascular complications but also exerts profound neurological impacts, complicating the management of these already complex conditions [11–16].

In an animal model of stroke, we have previously shown that stroke induces breathing disorders, including apnea and reduced breathing rates [17]. Additionally, we found that these breathing dysfunctions are associated with a decline in cognition [17].

Breathing is maintained by a negative feedback regulatory system designed to maintain blood gas homeostasis (i.e., obtain 02} and eliminate CO2) [18]. The retrotrapezoid nucleus (RTN), located in the ventral medullary region of the brainstem, contains specialized glutamatergic neurons that are pivotal for C02 sensing and the regulation of breathing rhythms [19, 20]. Despite the high prevalence of breathing disorders in dementia and stroke, how these feedback mechanisms, particularly at the brainstem regulatory center level, are affected by these conditions remains unknown.

Transforming Growth Factor Beta (TGF-β) is a cytokine that is part of the large transforming growth factor family. It exists in four latent isoforms–TGF-β1, TGF-β2, TGF-β3, and TGF-β4-which become active after proteolytic cleavage and binding to the type II TGF-β receptor. Increased levels of TGF-β have been detected in the brains of dementia patients. Interestingly, overexpression of TGF-β1 in astrocytes leads to perivascular gliosis and a higher amyloid burden around cerebral blood vessels [19, 21]. Additionally, activated astrocytes upregulate TGF-β signaling during ischemic stroke, promoting reactive gliosis and scar formation. We have previously shown that glial scar formation is detrimental during chronic post-stroke recovery, as it may reduce neurogenesis and angiogenesis in the infarct region. The effect of TGF-β on astrocytes outside the infarct region remains to be elucidated.

The exact mechanism that leads to breathing disorders in CAA and concurrent stroke and CAA is not clear. In this study, we examined the histology of the brainstem RTN in CAA mice. Furthermore, we tested the hypothesis that astrocytic TGF-β signaling at the RTN contributes to breathing instability and cognitive decline in CAA alone and in CAA with stroke.

## Materials and Methods

### Animals

Adult (13–14 months old) male Tg-SwDI (CAA model) mice and age/sex-matched C57BL/6 WT mice were bred in-house from Jackson Laboratories and used in all the experiments. Mice were kept in a climate-controlled vivarium, 5 per cage, on a 12-hour light/dark cycle with unrestricted access to food and water. Each mouse in a cage was randomly assigned a number from 1 to 5 by a lab member not involved in the surgical procedure. A random number generator was then used to assign each number to either the control or treatment groups.

### Animal Ethics declaration

All experiments were performed according to NIH guidelines for the care and use of animals in research and under protocols approved by the University of Texas Health Science Center Houston Institutional Animal Care and Use Committee.

### Stereotaxic Injections into the RTN

Mice were anesthetized and positioned in a stereotaxic apparatus. Lentiviral particles encoding gfa2-gRNA-miR124T/CMV-Cas9 (astrocytic specific, by GEG-Tech France) or control lentivirus were injected bilaterally into the retrotrapezoid nucleus (RTN). The lentivirus was diluted to a concentration of 2×109 TU/ml, and a volume of 300 nL was delivered using a Hamilton needle at coordinates 1.0 mm lateral to the midline, 5.6 mm caudal to bregma, and 5.6–5.7 mm ventral to the pial surface of the cerebellum bilaterally, following previously established methods [22, 23]. The injections were administered at a rate of 300 nL/min, with a total volume of 300 nL per hemisphere. Mice were tested 6 weeks after the onset of stereotaxic injection for behavior and plethysmography, then sacrificed for immunohistochemical (IHC) and molecular analysis.

### Middle Cerebral Artery Occlusion

Tg-SwDI and WT controls (C57BL/6) mice were subjected to Middle Cerebral Artery Occlusion (MCAO) to induce focal transient ischemic stroke as previously described [17, 24]. Briefly, mice were anesthetized with a mixture of isoflurane (5% for induction, 1.5–2% for maintenance) in room air. After subcutaneous injection of 0.25% Bupivacaine, a midline neck incision was made to expose the common carotid artery (CCA), external carotid artery (ECA), and internal carotid artery (ICA). A monofilament nylon suture was inserted into the ICA to block the middle cerebral artery. After 60 minutes of occlusion, reperfusion was established by suture removal. Four mice were excluded due to premature death following the surgery. Mice underwent stereotaxic injections three days after the onset of stroke, with either lentiviral particles for knocking down TGF-βR2 or vehicle control. Mice were tested for plethysmography and behavior tests, and then sacrificed 6 weeks after the stroke for IHC and molecular analysis.

### Whole-body Plethysmography

Breathing patterns were assessed using a whole-body plethysmography chamber designed for mice as we have previously published [17]. Mice were placed in the chamber and allowed to acclimate for 30 minutes before recording. Respiratory parameters such as tidal volume, respiratory rate, and minute ventilation were recorded for 20 minutes after 1-hour acclimation. To quantify apneic events in the plethysmography recordings, we adopted a dual-criterion approach. First, an apneic event was defined as a pause in breathing that lasted for at least twice the duration of the average breath during the baseline recording period, in accordance with established methods [17]. Second, any breathing period considered for apnea calculation needed to be at least 0.5 seconds in duration, as suggested by previous research [25]. Due to limitations in the accuracy of automated apnea detection from the recording software, all apneas were manually counted. We standardized the manual counting by selecting consistent time intervals for each subject, typically ranging from 5 to 10 minutes. The average number of apneas during these intervals was then calculated to yield a rate of apneas per minute. This approach was consistently applied across all subjects and strains.

### Barnes Maze

Mice underwent Barnes Maze to assess hippocampal-dependent spatial memory, as previously described [26, 27]. The maze consisted of a circular flat platform with 20 evenly spaced holes around the perimeter, one of which has a dark secure escape chamber underneath. The maze was lit with Bright lights, and a white noise machine was turned on during the trials. Mice were trained for 3 consecutive days to locate the escape box positioned underneath one of the holes. Each mouse had a maximum of 90 seconds to find the escape hole, and had 4 trials per training day. The maze was cleaned thoroughly after each mouse with 70% isopropyl alcohol to eliminate the odor cues. Latency to find the escape hole and the number of errors were recorded for each trial. On the fourth day, a test trial was performed to evaluate memory retention.

### Novel Object Recognition Test (NORT)

For testing nonhippocampal-mediated memory, mice were tested using Novel Object Recognition Test (NORT), as previously described [28]. Briefly, mice were put in an open field arena for a 5-min habituation period on day 1. On day 2, mice were placed in the same arenas with two identical objects and left to explore both objects. On the third day, one of the familiar objects were switched with a novel object. Testing stopped in all trials after each mouse reached a threshold of 30 seconds total exploration time between the two objects, and all trials was recorded and analyzed by EthoVision. The percentage of time spent with the novel object was calculated

### Immunohistochemistry (IHC)

Mice were deeply anesthetized and transcardially perfused with ice-cold phosphate-buffered saline (PBS), followed by 4% paraformaldehyde (PFA), as previously described [24]. The brainstems were extracted, post-fixed in PFA for 24 hours, and then cryoprotected in a 30% sucrose solution containing 0.02% sodium azide for 48 hours. The brainstems were then sectioned into 30-micron slices using a sliding microtome. The sections were washed with PBS and permeabilized for 15 minutes in 0.3% Triton X-100 in PBS. They were then blocked for 1 hour in a blocking solution comprising 5% donkey serum and 1% bovine serum albumin (BSA) in 0.3% Triton X-100. Following this, the sections were incubated overnight at 4°C with primary antibodies against GFAP (1:200, CST, Cat# 12389S) and TGF-βR2 (1:200, Abcam, Cat# ab186838) in the blocking solution. After incubation, the sections were washed with PBS and then incubated with the appropriate secondary antibody (1:1000, Abcam, anti-Goat Cat# ab150132, anti-Rabbit Cat# ab150073) for 1 hour in the dark at room temperature. Finally, the sections were mounted on slides, counterstained with DAPI, and coverslipped. For Negative controls, Sections were processed without primary antibodies to ensure specificity of the secondary antibodies and to rule out non-specific binding. Imaging was performed using the Leica Thunder Imaging System, and quantification was conducted using Fiji software version 2.15.1.

### Statistical Analysis

Statistical analysis and visualization were performed using Python 3.9.18 with the SciPy, numpy, Statsmodels, and Matplotlib packages [29–31]. Data are presented as means ± SEM. The significance level was set to 0.05, with significance indicated as follows: *P< 0.05, **P< 0.01, ***P < 0.001, and ****P <0.0001.

Normality of the data was assessed using the Shapiro-Wilk test. The Mann-Whitney U test was employed for statistical comparisons if the data did not meet the normality assumption (p < 0.05). Otherwise, a t-test was utilized. Throughout the analysis, investigators were blinded to the treatment groups.

## Results

### CAA Mice Show Disordered Respiratory Functions at Baseline

**Figure 1:**
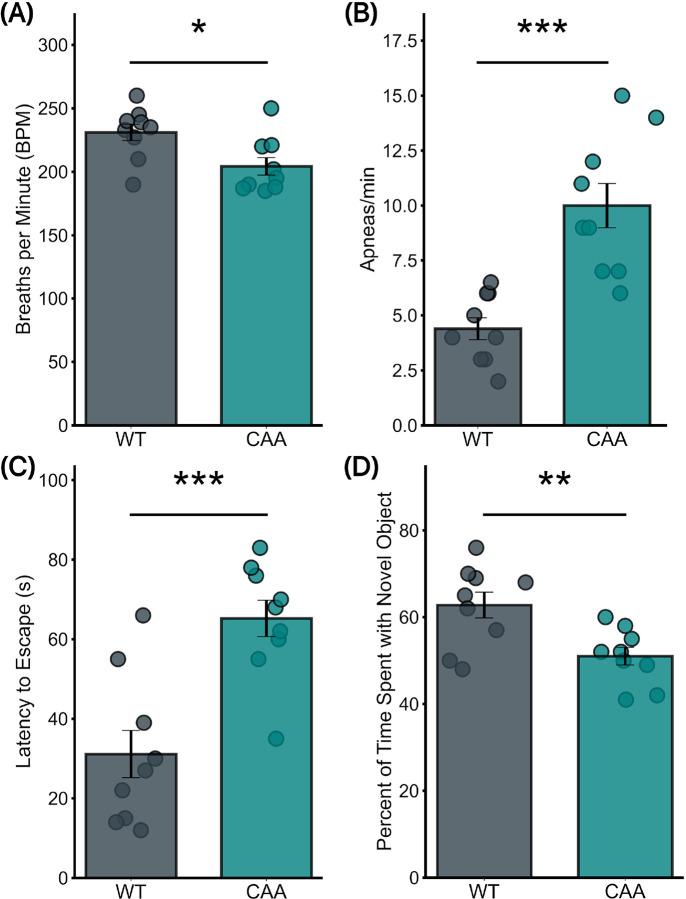
Baseline differences in physiological and cognitive parameters between naive CAA and WT mice (a) Respiratory frequency analysis shows a significant decrease in CAA mice compared to WT mice (Mean: 204±6.9 vs. 231±6.4; p=0.016). (b) Apnea rate comparison reveals a significant increase in CAA mice versus WT mice (Mean: 10±1 vs. 4.4±0.5; p=0.0002). (c) Barnes Maze test results demonstrate significantly poorer performance in CAA mice compared to WT mice (Mean: 65±4.56 vs. 31±5.95; p=0.0006). (d) Novel Object Recognition Test (NORT) outcomes indicate reduced cognitive engagement in CAA mice versus WT mice (Mean: 51±2 vs. 62.8±3; p=0.007). All groups consist of 9 mice each.

In cerebral amyloid angiopathy (CAA) mice without stroke, a significant decrease in respiratory rates was observed compared to wild-type (WT) controls (Fig. 1A). The CAA group exhibited an average respiratory rate of 204 ± 6.9 breaths per minute, significantly lower than the 231 ± 6.4 breaths per minute observed in WT mice (P = 0.016). Regarding apneic episodes, CAA mice demonstrated a significantly increased apnea frequency compared to WT mice (Fig. 1B), with mean values of 10 ± 1 and 4.4 ± 0.5 apneic episodes per minute respectively (p = 0.0002).

### CAA Mice Show Impaired Cognitive and Memory Impairment

In the Barnes Maze test, CAA mice exhibited significant impairment in spatial learning and memory (Fig. 1C). They showed longer latencies to locate the escape box, averaging 65 ± 4.56 seconds, versus 31 ± 5.95 seconds in WT mice (p = 0.0006).

In the NORT test, CAA mice showed a significant reduction in recognition memory compared to their WT counterparts (Fig. 1D), with a mean percentage of time spent with the novel object equal to 51 ± 2% and 62.8 ± 3% respectively (p = 0.007).

### CAA Mice Show Baseline Increases in Levels of TGF-βR2 and GFAP in the RTN

**Figure 2:**
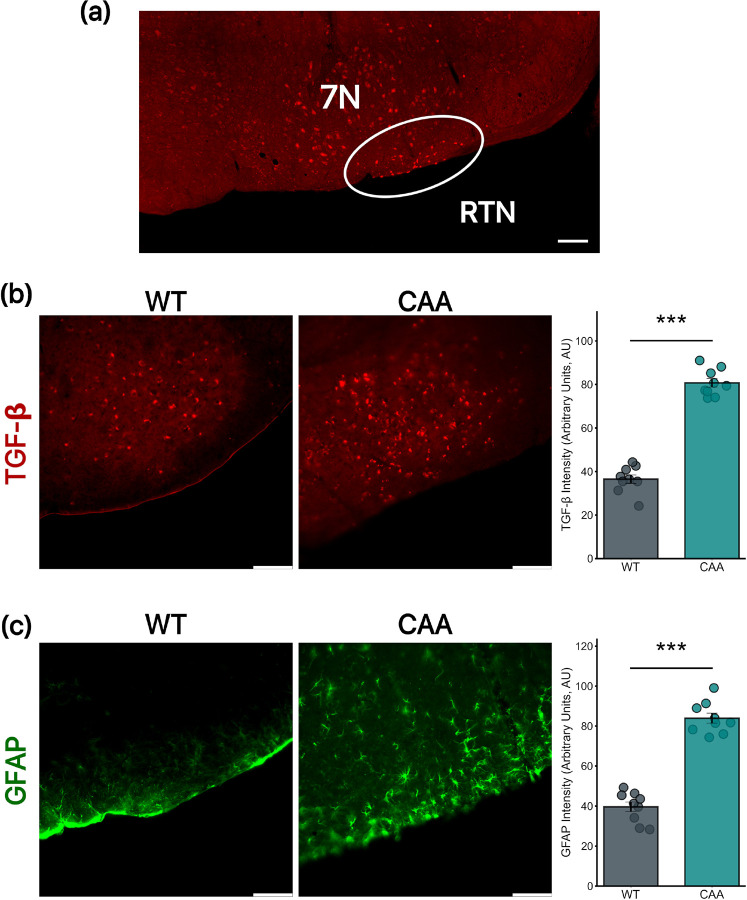
Localization and Comparative Analysis of RTN, TGF-βR2, and GFAP in the Brain Stem of WT and CAA Mice (a) Histological representation of the RTN location within the brainstem, illustrating the specific area analyzed in both WT and CAA mice, in relation to the 7N. (b) TGF-βR2 expression is significantly increased in CAA mice compared to WT mice (Mean = 36.48±1.93 vs. 80.71±1.94, p = <0.0001). (c) GFAP expression is significantly elevated in CAA mice compared to WT mice (Mean = 39.64±2.38 vs. 80.71±1.94, p = <0.0001). All groups consist of 9 mice each. Scale bar = 100 μm.

IHC analysis of TGF-βR2 expression in the brainstem of WT and CAA mice revealed a significant increase in TGF-βR2 levels in CAA mice compared to WT mice (Fig. 2B). The mean TGF-βR2 expression in WT mice was 36.48 ± 1.93, while in CAA mice, it was 80.71 ± 1.94. Similarly, GFAP expression, a marker for reactive astrocytes, was significantly elevated in CAA mice compared to WT mice (Fig. 2C). The mean GFAP expression in WT mice was 39.64 ± 2.38, whereas in CAA mice, it was 80.71 ± 1.94.

### Lentivirus-Induced TGF-βR2 KD in RTN Improved Respiratory function in CAA mice

**Figure 3:**
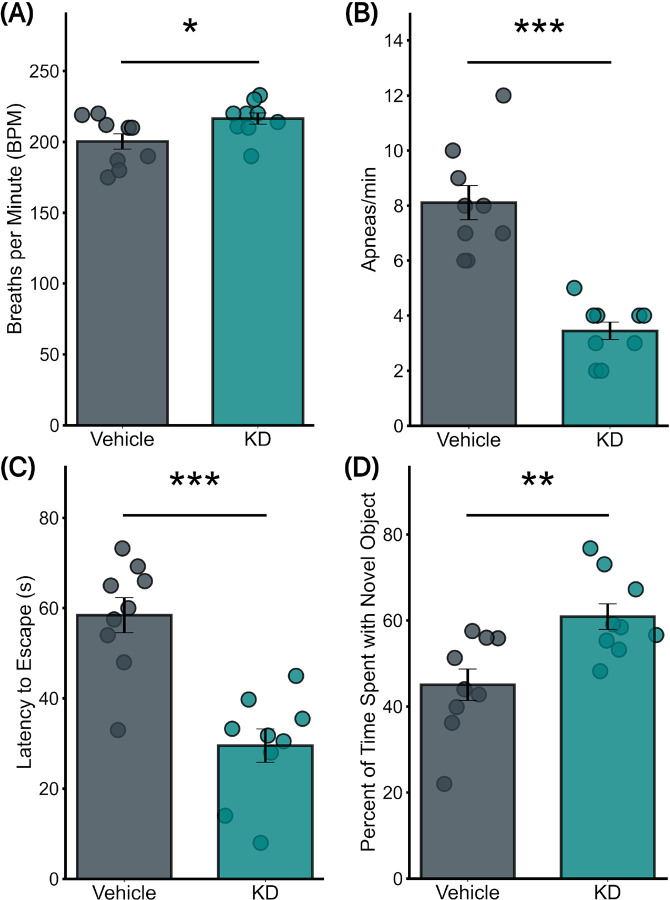
Impact of TGF-βR2 KD on CAA Mice’s Respiratory function and cognition (a) Respiratory rate comparison showing a significant increase in the KD group versus the vehicle group (Mean: 216±4 vs. 200±5; p=0.04). (b) Apnea rate analysis reveals a substantial decrease in the KD group versus the vehicle group (Mean: 3.4±0.3 vs. 8±0.6; p<0.0001). (c) Barnes Maze test results demonstrate significant cognitive improvements in KD-treated mice compared to vehicle-treated mice (Mean: 29.5±3.7 vs. 58.4±3.9; p=0.0001). (d) Novel Object Recognition Test (NORT) outcomes highlight enhanced engagement with novel objects in KD-treated mice versus vehicle-treated mice (Mean: 61±2.98 vs. 45±3.64; p=0.006). All groups consist of 9 mice each.

In CAA mice treated with lentivirus-induced TGF-βR2 KD, significant respiratory improvements were observed 6 weeks after injection. There was a remarkable decrease in average apnea episodes per minute in the TGF-βR2 KD group, dropping to 3.4 ± 0.3 from 8 ± 0.6 in the vehicle-treated group (p <0. 0001) (Fig. 3B). Furthermore, the respiratory rate in CAA mice also showed significant improvement post-TGF-βR2 KD, with the mean respiratory rate increasing from 200 ± 5 breaths per minute in the vehicle group to 216 ± 4 in the KD group (p = 0.04) (Fig. 3A).

### Lentivirus-induced TGF-βR2 KD improved NORT and Barnes maze Performance in CAA mice

A marked improvement in cognitive performance was observed in NORT in CAA mice following RTN-specific injection of a lentivirus to achieve TGF-PR2 knockdown (Fig. 3D). The TGF-βR2 KD group exhibited a significantly higher recognition, with a mean percentage of time spent with the novel object equal to 61 ± 2.98%, against 45 ± 3.64% in the vehicle-treated group (p = 0.006).

In the Barnes Maze test, CAA mice with lentivirus-induced TGF-βR2 KD exhibited significant improvements in spatial learning and memory (Fig. 3C). The TGF-βR2 KD group in CAA mice demonstrated enhanced maze navigation abilities, achieving an average escape time of 29.5 ± 3.7 seconds, notably shorter than the 58.4 ± 3.9 seconds in the vehicle group (p = 0.0001).

### Lentivirus-Induced TGF-βR2 KD Reduced Levels of TGF-βR2 and GFAP Expression in CAA Mice in RTN

To investigate the effects of targeted knockdown of transforming growth factor-beta receptor II (TGF-PR2) in cerebral amyloid angiopathy (CAA) mice, we conducted immunohistochemical analyses focusing on TGF-βR2 and glial fibrillary acidic protein (GFAP) expressions.

**Figure 4:**
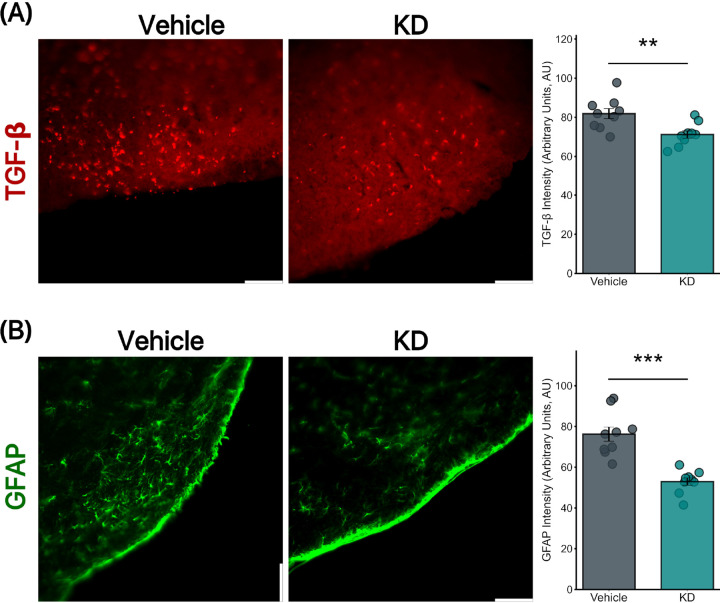
TGF-βR2 KD reduced GFAP in CAA mice (a) TGF-βR2 abundance is reduced in CAA TGF-βR2 KD mice (Right) compared to CAA Vehicle-treated mice (Left) (Mean: 81.9±2.56 vs. m71.180±1.85; p=0.006). (b) GFAP signaling, indicating reactive astrocytic activity, is decreased in CAA TGF-βR2 KD mice (Right) compared to CAA Vehicle-treated mice (Left) (Mean: 81.9±2.56 vs 71.180±1.85, p<0.0001). All groups consist of 9 mice each. Scale bar = 100 μm.

IHC staining for TGF-βR2 in CAA mice revealed significant differences between the vehicle-treated group and the TGF-βR2 knockdown (KD) group. The vehicle group displayed a mean TGF-βR2 expression level of 81.9 ± 2.56, while the KD group exhibited a reduced mean expression level of 71.180 ± 1.85 (Fig. 4A). For GFAP analysis in the RTN, the vehicle-treated mice had a mean GFAP expression of 76.2 ± 3.46, whereas the KD mice demonstrated a significantly lower mean expression of 52.94 ± 1.79 (Fig. 4B).

### Lentivirus-Induced TGF-βR2 KD Increased Respiratory Functions in CAA Mice with Concurrent Stroke

**Figure 5:**
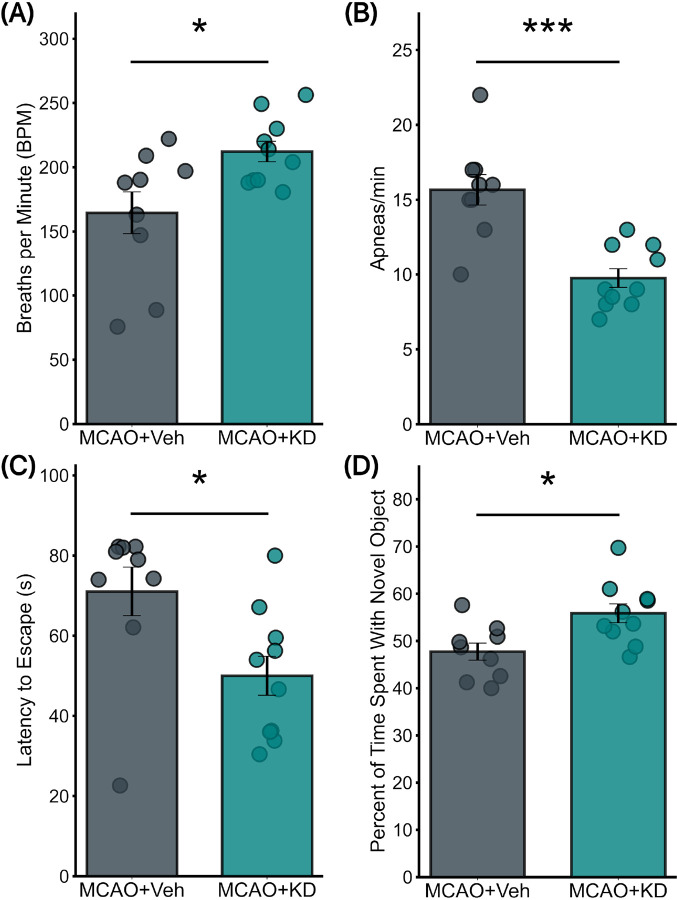
Impact of TGF-βR2 KD on Respiratory and Cognitive Parameters in CAA Mice with concurrent MCAO (a) Respiratory frequency analysis demonstrates a significant increase in MCAO mice treated with TGF-βR2 KD compared to those treated with vehicle (Mean: 212±8 vs. 165±16; p=0.02). This highlights the improved respiratory function in the KD-treated group. (b) Apnea rate comparison shows a significant reduction in MCAO mice treated with TGF-βR2 KD versus those treated with vehicle (Mean: 9.8±0.6 vs. 15.7±1; p=0.0002). This suggests a beneficial effect of TGF-βRII KD on reducing apnea episodes. (c) Barnes Maze test results indicate a significant improvement in cognitive performance in MCAO mice treated with TGF-βR2 KD compared to vehicle-treated mice (Mean: 50±4.9 vs. 71±6; p=0.016, Mann-Whitney U test). (d) Novel Object Recognition Test (NORT) outcomes reveal enhanced cognitive engagement in MCAO mice treated with TGF-βR2 KD compared to those treated with vehicle (Mean: 56±2 vs. 48±1.8; p=0.01). (N=9 for MCAO + Veh and 10 for MCAO+KD)

We then studied the effect of RTN TGF-βR2 KD in CAA mice with concurrent stroke. In CAA mice with MCAO stroke, the introduction of lentivirus-induced TGF-βR2 KD significantly improved respiratory functions assessed 6 weeks after stroke. The MCAO + TGF-βR2 KD group exhibited a significant increase in respiratory rate (Fig. 5A), averaging 212 ± 8 breaths per minute compared to 165 ± 16 breaths per minute in the MCAO + Vehicle group (p = 0.02). Additionally, this group showed a marked reduction in apnea frequency, averaging 9.8 ± 0.6 episodes per minute, significantly lower than the 15.7 ± 1 episodes per minute observed in the vehicle group (p = 0.0002) (Fig. 5B).

### TGF-βR2 KD Enhanced Cognitive Performance in CAA with Concurrent MCAO

Consistent with the respiratory improvements, cognitive benefits were also observed in the CAA mice with MCAO after TGF-βR2 KD. The NORT demonstrated significant enhancements in recognition memory, with the MCAO + TGF-βR2 KD group spending an average of 56 ± 2% of the time with the novel object, compared to 48 ± 1.8% in the vehicle group (p = 0.01) (Fig. 5D). Furthermore, the Barnes Maze test results indicated improved spatial learning and memory (Fig. 5C), with these mice navigating the maze in an average time of 50 ± 4.9 seconds, significantly faster than the 71 ± 6 seconds recorded for the vehicle group (p = 0.016, Mann-Whitney U test due to normality failure in the vehicle group).

### TGF-βR2 Knockdown Reduced TGF-βR2 and GFAP Expression in the RTN of Mice with Stroke

**Figure 6:**
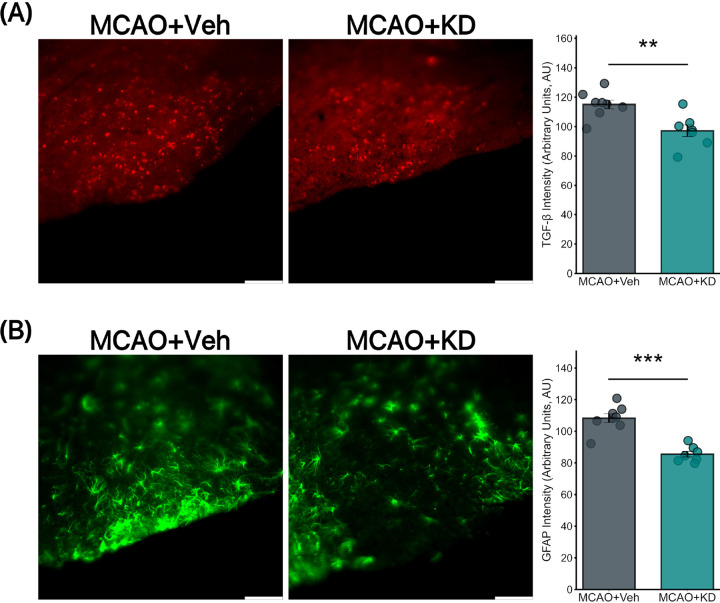
TGF-βR2 KD reduced GFAP in CAA mice with stroke Immunohistochemistry analysis was performed to evaluate the effects of MCAO CAA TGF-βR2 treatment on KD mice. (a) TGF-βR2 abundance is significantly reduced in MCAO CAA TGF-βR2 KD mice (Right) compared to MCAO CAA Vehicle-treated mice (Left) (115±2.95 vs 97.18±3.931, p=0.005). (b) GFAP signaling, indicative of reactive astrocytic activity, is significantly decreased in MCAO CAA TGF-βR2 KD mice (Right) compared to MCAO CAA Vehicle-treated mice (Left) (Mean: 108.3±2.744 vs 85.5±1.768, p<0.0001). (N=8 for MCAO + Veh and 7 for MCAO+KD). Scale bar = 100 μm.

IHC analysis of TGF-βR2 expression in the brainstem of MCAO mice revealed a significant reduction in TGF-βR2 levels in the TGF-βR2 knockdown (KD) group compared to the vehicle-treated group (Fig. 6A). The mean TGF-βR2 expression in the MCAO + Veh group was 115 ± 2.95, while in the MCAO + KD group, it was 97.18 ± 3.931. Similarly, GFAP expression was significantly decreased in the MCAO + KD group compared to the MCAO + Veh group. The mean GFAP expression in the MCAO + Veh group was 108.3 ± 2.744, whereas in the MCAO + KD group, it was 85.5 ± 1.768. (Fig. 6B).

## Discussion

Our work has yielded several significant new findings: First, we revealed brainstem RTN gliosis in CAA mice. Second, we discovered respiratory disorders in CAA mice in experimental models. Third, our data showed that TGF-β signaling is significantly increased in the brainstem RTN, and this increased signaling is responsible for gliosis and respiratory disorders in CAA. Knocking down the receptor improved breathing and cognitive function in CAA mice without stroke. Fourth, in CAA mice with MCAO stroke, we found that knocking down TGF-β receptor II reduces gliosis in the RTN, stabilizes breathing, and reduces cognitive deficits. Our study is the first to report a causal link between respiratory disorder and cognitive decline in CAA alone and CAA with stroke.

Respiratory activity is maintained by a negative feedback system designed to maintain blood gas homeostasis. Central and peripheral chemoreceptors form the feedback portion of the control loop by adjusting the rate and depth of breathing in response to changes in tissue CO_2_/H+ and O_2_ [19, 32]. Central chemoreceptors communicate directly with the central pattern generator (CPG), known as the Botzinger Complex [33]. In coordination with key respiratory neuronal populations such as the NTS and the RTN, the pacemaker and non-pacemaker cells of the Botzinger Complex control rhythmic respiratory activity [19, 33]. To the best of our knowledge, this is the first report of pathological changes in the RTN in dementia models.

Under pathological conditions such as stroke, astrocytes become reactive, markedly increasing the expression of glial fibrillary acidic protein (GFAP), the hallmark signature of reactive gliosis, in response to intercellular signaling molecules including IL-6, TNF*α*, TGF-β, INF*γ*, and IL-10. Reactive astrocytes have the potential to alter their function, which can be both beneficial and detrimental to the brain. In pathological conditions, reactive astrocyte processes overlap, forming a persistent scar [19].

Reactive gliosis, indicated by GFAP-positive astrocytes, was markedly evident in CAA mice and CAA mice with stroke. It is worth noting that GFAP immunoreactivity is the current hallmark of astrocyte reactivity, and its detection may be extremely limited in quiescent astrocytes [34]. Brainstem astrogliosis may disrupt breathing via several mechanisms, including basement membrane fibrosis, purinergic control of vascular tone, or stimulation of neuronal activity [19, 35]. Glia scar formation at RTN may simply act as a physical barrier that prevents chemoreceptors from detecting changes in CO_2_/H^+^. Alterations in basement membrane fibrosis induced by reactive gliosis may also inhibit both neuronal and astrocyte detection of CO_2_/H^+^ [19], thereby disrupting breathing patterns. However, the detailed biochemical and electrophysiological mechanisms by which RTN gliosis contributes to respiratory dysfunction remain to be fully explained.

TGF-β levels are increased in both stroke and dementia and are key inducers of gliosis. Binding with the TGF-β receptor initiates the SMAD signaling pathway, resulting in the phosphorylation of SMAD proteins. The RSMAD/coSMAD complex then translocates to the nucleus, binding to various transcriptional promoter sites and altering the expression of a variety of genes [19, 36]. We demonstrated that increased TGF-β signaling in the brainstem plays a causal role in respiratory disorders in CAA and CAA with concurrent stroke, likely by inducing gliosis and disrupting chemoreception in the breathing control center.

We showed that improving breathing is effective in reducing cognitive impairment in both CAA alone and CAA with stroke. It is possible that breathing disorders induce intermittent hypoxia in mice, as we previously demonstrated in a stroke model [10]. Intermittent hypoxia may then increase amyloid burden in the brain through various mechanisms. For example, breathing disorders lead to disrupted sleep, which negatively impacts the glymphatic flow, a key mechanism for amyloid beta clearance in the brain [37]. Chronic intermittent hypoxia may also increase amyloid beta production [38]. The exact mechanisms require further investigation in animal models.

There are several limitations to our study. First, we only used male mice. Biological variables such as sex are crucial considerations for translational studies, and female mice will used to study how breathing is regulated in CAA and CAA with stroke in the future. Second, direct brainstem injection is challenging for translation into clinical practice. We are aware of this and we will conduct experiments in which mice are treated via the oral route with TGF-β receptor antagonists in both sexes.

In conclusion, our research is the first to demonstrate brainstem gliosis in CAA mice, establishing a direct link between gliosis and both respiratory and cognitive dysfunctions. We have shown that targeting TGF-β signaling not only mitigates these impairments but also improves overall outcomes in CAA models, both with and without stroke.

## Data Availability

Data is available from the corresponding author upon reasonable request.
